# Health Services Use and Costs in Individuals with Autism Spectrum Disorder in Germany: Results from a Survey in ASD Outpatient Clinics

**DOI:** 10.1007/s10803-021-04955-4

**Published:** 2021-03-17

**Authors:** Juliana Höfer, Falk Hoffmann, Michael Dörks, Inge Kamp-Becker, Charlotte Küpper, Luise Poustka, Stefan Roepke, Veit Roessner, Sanna Stroth, Nicole Wolff, Christian J. Bachmann

**Affiliations:** 1 Department of Health Services Research, Carl von Ossietzky University Oldenburg, Oldenburg, Germany; 2 Department of Child and Adolescent Psychiatry, Psychosomatics and Psychotherapy, Philipps University Marburg, Marburg, Germany; 3 Department of Psychiatry, Campus Benjamin Franklin, Charité - Universitätsmedizin Berlin, Berlin, Germany; 4 Present Address: Department of Psychology, Humboldt-Universität zu Berlin, Berlin, Germany; 5 Present Address: Department of Child and Adolescent Psychiatry, University Medical Center Göttingen, Göttingen, Germany; 6 Department of Child and Adolescent Psychiatry, Medical Faculty of the Technical University Dresden, Dresden, Germany; 7 Department of Child and Adolescent Psychiatry, University of Ulm, Ulm, Germany; 8 Department of Child and Adolescent Psychiatry and Psychotherapy, Medical Faculty Mannheim, Central Institute of Mental Health, Heidelberg University, Mannheim, Germany; 9 Department of Child and Adolescent Psychiatry, Medical University of Vienna, Vienna, Austria

**Keywords:** Adolescents, Adults, Children, Costs, Germany, Health services

## Abstract

Autism spectrum disorders (ASD) are associated with high services use, but European data on costs are scarce. Utilisation and annual costs of 385 individuals with ASD (aged 4–67 years; 18.2% females; 37.4% IQ < 85) from German outpatient clinics were assessed. Average annual costs per person were 3287 EUR, with psychiatric inpatient care (19.8%), pharmacotherapy (11.1%), and occupational therapy (11.1%) being the largest cost components. Females incurred higher costs than males (4864 EUR vs. 2936 EUR). In a regression model, female sex (Cost Ratio: 1.65), lower IQ (1.90), and Asperger syndrome (1.54) were associated with higher costs. In conclusion, ASD-related health costs are comparable to those of schizophrenia, thus underlining its public health relevance. Higher costs in females demand further research.

**Supplementary Information:**

The online version contains supplementary material available at 10.1007/s10803-021-04955-4.

Autism spectrum disorder (ASD) is a pervasive neurodevelopmental disorder with a prevalence of up to 1%, which usually manifests in early childhood (Lord et al., 2020). According to the DSM-5 and ICD-11, the core symptoms of ASD are profound deficits in the domains of social interaction and communication, and restricted, repetitive, and inflexible patterns of behaviour and/or interests. About half of individuals with ASD have intellectual disability. Rates of co-occurring somatic or psychiatric disorders also are high in this population (Fortuna et al., 2016; Hossain et al., 2020; Muskens et al., 2017), leading to increased mortality (Hirvikoski et al., 2016).

As a consequence, individuals with ASD often require a broad range of support services, e.g. special education (Bürki et al., 2021), diagnostic and therapeutic health services (Jobski et al., 2017), social services (Fortuna et al., 2016; Kamp-Becker et al., 2010), and supported employment (Vogeley et al., 2013). This extensive service use yields significant societal costs: A fairly recent modelling study estimated the lifetime costs of supporting an individual with ASD without intellectual disability as US$1.4 million (United States of America (US)), and £920,000 (United Kingdom (UK)), respectively (Buescher et al., 2014).

In order to ensure appropriate planning of the afore-mentioned services and allocation of resources, it is key to have a clear understanding of the main costs associated with ASD. To date, the majority of research on ASD-related costs stems from the US: A recent review evaluated 48 relevant publications, of which more than half were from the US (Rogge & Janssen, 2019). Regarding Europe, only studies from the UK, Sweden and the Netherlands were available. For Germany, there exists only a cost-of-illness model for individuals with ASD without an intellectual disability (Bachmann et al., 2013). What most of these publications have in common is that they either present theoretical cost-of-illness models (e.g. Buescher et al. (2014)) or rely on secondary data, usually from health insurance funds, e.g. Cidav et al. (2013). Both approaches potentially affect the accuracy of the resulting findings, either through imprecision regarding the underlying estimates (in cost-of-illness models), or through uncertain validity of the coded diagnoses (in secondary data studies).

In contrast, studies on ASD-related costs based on “real-life” service use data (e.g. Barrett et al. (2015)) are scarce—to date there exist only four studies (two from the UK, one from Ireland, and one from the US), with none of these reporting data on adults with ASD.

Given the lack of real-life user data from European countries on service use and related costs in individuals with ASD—especially in adults, this study aimed to estimate health service use and associated costs in a validly diagnosed sample of German children, adolescents and adults with ASD with and without intellectual disability.

## Methods

This research study was conducted within the framework of the ASD-Net, a German clinical and research network that focuses on key challenges in ASD diagnostics, therapy and health service research (Kamp-Becker et al., 2017).

### Recruitment and Participants

Study data was collected in four German ASD outpatient clinics from patients with a confirmed diagnosis of a pervasive developmental disorder according to ICD-10 (F84.0, F84.1, F84.5, F84.8, and F84.9). All participants had been diagnosed by experienced clinicians using the current diagnostic gold standard in ASD, including the Autism Diagnostic Observation Schedule (ADOS), and the Autism Diagnostic Interview-Revised (ADI-R) (Bölte et al., 2006; Hayes et al., 2018; Rühl et al., 2004). Participants’ IQ had been assessed using the German versions of the following instruments: Wechsler Intelligence Scale for Children (WISC-R (Tewes, 1983), WISC-III (Tewes et al., 1999), WISC-IV (Petermann & Petermann, 2011)), Wechsler Adult Intelligence Scale (WAIS-R (Tewes, 1991), WAIS-III (von Aster et al., 2006), WAIS-IV (Petermann, 2012)), Wechsler Preschool and Primary Scale of Intelligence (WPPSI-III (Petermann et al., 2009)), Kaufman Assessment Battery for Children (Melchers & Preuß, 2009), Wortschatztest (Schmidt & Metzler, 1992), Raven’s Standard Progressive Matrices (Horn, 2009), and Raven’s Coloured Progressive Matrices (Bulheller & Häcker, 2002). While intellectual disability is defined as an IQ < 70, some studies on ASD have included “borderline intellectual functioning” (IQ = 71–84) (Baker & Blacher, 2020). Yet, according to ICD-10 (Dilling & Freyberger, 2012), an IQ between 71 and 84 is defined as a learning disability. Referring to this definition, in the current study IQ levels were dichotomised into learning disability/ intellectual disability (IQ < 85) vs. no learning disability/ intellectual disability (IQ ≥ 85).

### Questionnaire and Data Collection

Sociodemographic data (including age, sex, and educational attainment) and health service utilisation was assessed through a questionnaire which included a modified version of the Client Service Receipt Inventory (CSRI). The CSRI is a well-established semi-structured interview, in which participants are asked about health services used over the preceding 12 months (Beecham & Knapp, 2001). The instrument can be administered to the service user face to face or by post for self-completion (Patel et al., 2005).

All people who had received any services in one of the four ASD clinics were invited to participate. 1419 survey documents were sent to patients with ASD or their parents/legal guardians (of these, 125 documents could not be delivered due to a wrong address). Patients and caregivers, respectively, received the questionnaire via postal mail and were asked to consent both to the study, and to the pseudonymised linkage of study data with their clinical data (e.g. clinical diagnosis, level of intellectual functioning, ADOS comparison score). Specific data on socioeconomic status and race/ethnicity was not recorded. For participants under 18 years of age, parents/legal guardians filled in the questionnaire. The questionnaire for adults with ASD was directly addressed to these. We assumed that the majority of adult participants with an IQ < 85 were assisted in filling in the questionnaire. 418 persons returned the questionnaire including a signed written consent form (response: 32.3%), of which 385 questionnaires could be evaluated. Characteristics of survey responders vs non-responders are reported in the results section.

Within the German health insurance system (Busse & Blumel, 2014), the majority of health services is largely free of charge for patients; including inpatient and outpatient health care, prescription medication, rehabilitation, non-physician practitioner services (e.g. speech therapy, occupational therapy), and—rather exceptional within Europe—psychotherapy (Signorini et al., 2017). Regarding patients with ASD, most health services (e.g. diagnostic procedures, follow-up examinations, medication, interventions for co-occurring disorders) are reimbursed by statutory health insurance funds. Nevertheless, interventions or support concerning the core symptoms of ASD are covered by integration support aid (“Eingliederungshilfe”) which is funded by social services. As these interventions are provided by different public and private institutions, comprise very diverse approaches (from facilitated communication to behavioural interventions and others), and are not part of the statutory health insurance system, we did not include them in our study.

CSRI items related to contacts with all types of healthcare professionals, and duration of all inpatient and rehabilitation treatment in the last 6 or 12 months, respectively. Outpatient drug prescriptions were assessed for the previous 4 weeks. All figures were extrapolated to a 12-month time period, under the assumption that the data were representative of the entire year.

Questionnaire data was entered by one person in an electronic Case Report Form (eCRF) created in OpenClinica® (OpenClinica Enterprise Version: 3.3), and was checked by another person.

### Estimation of Service Use Costs

Based on the CSRI data, annual service use costs for inpatient and outpatient medical care were calculated, and stratified by sex, age group, diagnostic category, ADOS symptom score, intellectual functioning (IQ < 85 vs. IQ ≥ 85), and educational level. The level of education was defined in accordance with the International Standard Classification of Education (ISCED) (UNESCO, 1997, 2012), and was classified into three categories: low (ISCED level 0–2B), medium (level 2A) and high education (level 3A). Referring to the German school system, low educational level complies with 9 years of schooling or leaving school without having acquired any school-leaving qualification. Medium educational level is equivalent to 10 years of schooling, and high educational level complies with 12 or 13 years of schooling and a school-leaving qualification, which opens access to higher educational institutions (Schneider, 2008; Schroedter et al., 2011).

Direct costs were calculated by multiplying each resource use item by an appropriate unit cost (macro-costing). All costs were assessed from the perspective of the social security system and were calculated in Euros based on prices of the year 2016, obtained from a number of sources (Table S1 in Additional File 1): Where possible, medical services, including consultations of medical practitioners and non-physician practitioners (physical therapy, occupational therapy, speech therapy), as well as hospital stays and rehabilitation were valued by unit costs according to the calculation of Bock et al. (2015). Costs for outpatient hospital care as well as consultations of child and adolescent psychiatrists and psychotherapists for children were estimated by experts, using EBM-Codes (Doctors’ Fee Scale within the German Statutory Health Insurance Scheme) and/or quarterly fixed amounts. In cases of fixed amounts, a maximum of four were charged for participants who stated more than three contacts per year. For adults, the cost of medication was estimated by means of defined daily doses (DDD) using the Drug Prescription Report 2017 (Arzneiverordnungs-Report, 2017) or previous issues for example if drugs were withdrawn from the market. For children, the DDD and thereby the costs were estimated using either the Drug Prescription Report or were calculated based on the information given in the summary of product characteristics of each drug. In the latter case, the DDD were assessed based on the assumption of a twelve years old child with a weight of 41.5 kg (50th age-adjusted percentile, www.pedz.de). Finally, daily drug costs for long-time therapy drugs were multiplied by 365.25.

### Data Analyses

Baseline data were analysed using descriptive statistics. Mean values and standard deviations for healthcare service use, stratified by type of service (inpatient services, other hospital-based services, outpatient services and non-physician practitioner services), were calculated separately for children (0–11 years), adolescents (12–17 years) and adults (≥ 18 years). To account for non-normality of cost data, average annual costs per individual and by type of service were estimated with 95% confidence intervals (95% CI), using the bias-corrected accelerated bootstrap method (Barker, 2005) based on 5000 replications. Additionally, in order to detect associations between potential predictors (sex, age, ASD diagnoses, intellectual functioning and ADOS comparison score) and total costs, a generalised linear model assuming a gamma distribution with a logarithmical link function was fitted to calculate cost ratios. All statistical analyses were performed with SAS, version 9.4 (SAS Institute, Cary, US). The underlying research data are available from the corresponding author upon reasonable request.

## Results

The baseline characteristics of the sample are presented in Table 1. Of all 385 study participants, 81.8% were male (mean age: 21.6 years) and 18.2% were female (mean age: 23.8 years). Overall, 50.9% of participants were aged 18 years or older. 37.4% had a learning disability or intellectual disability (IQ < 85). The most frequent diagnosis was childhood autism (42.3%), followed by Asperger syndrome (39.5%). 43.9% of the participating adults with ASD, and 57.7% of parents of children and adolescents with ASD had a high level of education. Survey responders and non-responders did not differ markedly in terms of age, gender, or IQ, but differed with regard to the ASD subgroups, with a higher proportion of individuals with Asperger syndrome in the responder group (39.5% vs. 24.7%).

**Table 1:** Baseline characteristics

Characteristic	Mean	SD (range)
Age, in years (n = 385)	22.0	12.2 (4–67)

	n	%
Sex (n = 385)		
Male	315	81.8
Female	70	18.2
Age groups, in years (n = 385)		
0–11	67	17.4
12–17	122	31.7
≥ 18	196	50.9
Diagnoses (n = 385)		
Childhood autism (F84.0)	163	42.3
Atypical autism (F84.1)	63	16.4
Asperger syndrome (F84.5)	152	39.5
Other (F84.8/.9)	7	1.8
Intellectual functioning (n = 340)		
No learning disability/intellectual disability	213	62.6
Learning disability/intellectual disability	127	37.4
ADOS comparison score (n = 332)		
Minimal to low (1–4)	59	17.8
Moderate (5–7)	152	45.8
High (8–10)	121	36.4
Level of education (adults; n = 189)		
Low/medium	106	56.1
High	83	43.9
Highest parental level of education (children; n = 189)		
Low/medium	78	41.3
High	111	58.7

Health services use by age and type of service are shown in Table 2. Amongst inpatient services, psychiatric/psychosomatic care was used most frequently (4.7% of all participants), while dentists/orthodontists (65.9%), general practitioners (47.2%) and paediatricians (31.3%) were the most frequently consulted outpatient services. Amongst non-physician practitioner services, occupational therapy (22.8%) was the leading treatment modality.

**Table 2:** Health services use, by age and type of service

Type of service	Unit	0–11 years				12–17 years				≥ 18 years				Overall			
		N	Mean	SD	Persons with ≥ 1 contact, in %	N	Mean	SD	Persons with ≥ 1 contact, in %	N	Mean	SD	Persons with ≥ 1 contact, in %	N	Mean	SD	Persons with ≥ 1 contact, in %
Inpatient services																	
Psychiatric or psychosomatic inpatient care	Days in previous 12 months	67	0.13	0.72	4.48	122	0.02	0.18	0.82	196	2.38	13.14	7.14	385	1.24	9.44	4.68
Non-psychiatric inpatient care		67	0.16	0.95	2.99	122	0.16	1.01	3.28	196	0.31	1.77	5.10	385	0.24	1.44	4.16
Inpatient rehabilitation centre		67	2.62	7.80	11.94	122	0.80	4.04	4.10	196	0.25	2.49	1.02	385	0.84	4.41	3.90
Other hospital-based services																	
Emergency department	Days in previous 12 months	67	0.48	1.05	19.40	122	0.11	0.53	4.92	196	0.43	1.37	13.78	385	0.34	1.12	11.95
Outpatient clinic		67	1.52	6.38	17.91	122	1.30	5.60	12.30	195	1.26	4.73	16.41	384	1.32	5.32	15.36
Day care unit		67	1.73	12.26	5.97	122	1.15	12.68	0.82	195	1.01	9.21	3.59	384	1.18	10.94	3.13
Outpatient services																	
General practitioner	Visits in previous 12 months	65	0.34	1.35	7.69	121	1.12	2.31	26.45	193	3.99	4.81	73.58	379	2.45	4.03	47.23
Paediatrician		65	3.29	3.09	75.38	120	2.23	3.54	50.00	195	0.31	1.73	5.13	380	1.43	2.92	31.32
Adult psychiatrist		66	0.03	0.25	1.52	122	0.21	1.85	2.46	193	1.89	5.16	27.98	381	1.03	3.92	15.22
Child and adolescent psychiatrist		64	0.97	3.55	17.19	121	1.02	2.79	22.31	196	0.47	3.65	4.59	381	0.73	3.39	12.34
Psychotherapist (for adults)		66	0.61	3.08	9.09	122	1.31	6.39	7.38	192	3.54	9.74	19.79	380	2.26	8.01	13.95
Psychotherapist (for children)		66	0.97	3.82	9.09	122	1.07	5.97	5.74	195	0.27	2.10	2.05	383	0.64	4.02	4.44
Centre for social paediatrics		66	0.33	1.09	10.61	122	0.13	0.56	5.74	195	0.02	0.20	1.03	383	0.11	0.58	4.18
Psychiatric outpatient clinic		65	0.03	0.25	1.54	121	0.13	1.12	2.48	195	0.27	1.83	2.56	381	0.18	1.46	2.36
ASD outpatient clinic		65	1.57	3.45	38.46	120	2.93	8.55	28.33	194	1.53	3.85	22.16	379	1.98	5.75	26.91
Dentist/Orthodontist		65	2.55	2.70	67.69	119	3.71	4.24	76.47	191	2.00	2.57	58.64	375	2.64	3.30	65.87
Ophthalmologist/Optician		65	0.68	1.08	30.77	121	0.91	1.53	34.71	195	0.57	1.35	21.03	381	0.70	1.37	27.03
ENT specialist/Hearing aid audiologist		65	0.52	1.74	13.85	121	0.28	1.35	6.61	195	0.47	1.33	14.87	381	0.42	1.41	12.07
Non-physician practitioner services																	
Speech therapy	Visits in previous 12 months	63	23.43	25.90	50.79	121	6.10	16.86	12.40	195	2.29	11.71	4.62	379	7.02	18.09	14.78
Occupational therapy		64	27.56	25.55	62.50	122	7.48	17.54	18.03	195	4.32	14.21	12.82	381	9.23	19.26	22.83
Physiotherapist		65	9.57	21.06	24.62	121	8.50	18.95	19.83	194	7.28	21.54	18.04	380	8.06	20.63	19.74
Prescription medication	Previous 4 weeks	67	–	–	17.91	122	–	–	36.07	196	–	–	44.39	385	–	–	37.14

The average annual costs for health services per individual are displayed in Table 3. The average annual cost per individual with ASD was 3287 (95% CI 2.81–4.14) EUR. Service use distinctly differed by sex, with females incurring higher costs than males (4864 EUR vs. 2936 EUR).

**Table 3:** Average annual costs per individual

	Mean, €	95% CI	
Sex			
Male	2936.39	2470.53	3962.18
Female	4864.00	3475.25	6984.55
Age groups, in years			
0–11	4177.01	3497.54	5000.22
12–17	2509.88	2065.93	3287.96
≥ 18	3466.21	2618.03	5071.10
Diagnoses			
Childhood autism (F84.0)	3205.76	2610.65	4220.33
Atypical autism (F84.1)	3716.22	2942.27	4911.17
Asperger syndrome (F84.5)	3208.21	2332.61	5400.41
Other (F84.8/.9)	3018.98	1433.40	4788.24
Intellectual functioning			
No learning disability/intellectual disability	2484.43	1962.97	3281.70
Learning disability/intellectual disability	4474.64	3447.86	6769.88
ADOS comparison score			
Minimal to low (1–4)	3008.24	2267.02	4553.08
Moderate (5–7)	3362.36	2708.78	4365.18
High (8–10)	2823.53	1957.02	5545.28
Level of education (adults)			
Low/medium	4421.76	3132.23	7415.05
High	2378.26	1508.96	4232.01
Highest parental level of education (children)			
Low/medium	2954.36	2451.60	3503.58
High	3133.88	2566.79	4002.02
Total	3286.86	2811.50	4140.95

Costs for children with ASD (4177 EUR) were higher than for adults (3466 EUR) or adolescents (2510 EUR). Patients with an IQ < 85 yielded higher costs than those with an IQ ≥ 85 (4475 EUR vs. 2484 EUR). Costs did not differ very much by diagnosis, with costs for individuals with Asperger syndrome being only marginally higher. Higher ADOS comparison scores were not associated with higher costs.

The different cost components are shown in Table 4, with inpatient psychiatric care (19.8% of total cost), pharmacotherapy (11.1%), and occupational therapy (11.1%) being the largest components.

**Table 4:** Average annual costs by type of service

Type of service	0–11 years			12–17 years			≥ 18 years			Overall			
	Mean, €	95% CI		Mean, €	95% CI		Mean, €	95% CI		Mean, €	95% CI		% Total costs
Inpatient services													
Psychiatric or psychosomatic inpatient care	70.43	7.83	219.12	8.60	0.00	25.79	1249.27	572.47	2800.83	650.97	317.32	1450.39	19.8
Non-psychiatric inpatient care	37.42	0.00	108.87	37.37	9.34	106.50	70.94	31.40	160.49	54.47	30.79	104.79	1.7
Inpatient rehabilitation centre	406.48	194.00	794.49	123.03	39.32	298.06	38.69	0.00	121.58	129.42	74.36	214.22	3.9
Other hospital-based services													
Emergency department	16.53	8.26	25.82	3.97	1.13	7.94	14.83	9.53	23.30	11.68	8.45	16.54	0.4
Outpatient clinic	76.58	27.03	223.72	65.14	32.16	150.08	146.12	89.56	251.86	106.90	71.54	164.68	3.3
Day care unit	256.52	22.11	1211.82	170.02	0.00	510.06	148.92	33.43	554.45	174.40	63.11	435.22	5.3
Outpatient services													
General practitioner	7.17	1.98	17.96	23.79	16.37	33.66	84.46	72.02	102.92	51.84	44.35	61.45	1.6
Paediatrician	119.08	94.26	148.12	80.78	61.49	108.81	11.13	5.19	25.49	51.59	42.26	64.01	1.6
Adult psychiatrist	1.38	0.00	6.78	9.69	0.75	42.48	85.74	59.68	128.53	46.77	32.67	70.45	1.4
Child and adolescent psychiatrist	101.16	53.12	172.64	142.68	99.06	201.65	33.03	15.41	61.66	79.30	60.52	105.05	2.4
Psychotherapist (for adults)	49.46	14.62	197.84	92.31	28.10	242.15	289.04	192.97	427.28	184.27	125.52	262.97	5.6
Psychotherapist (for children)	84.61	26.85	200.94	92.97	30.04	238.86	23.27	6.24	66.22	56.04	28.32	101.60	1.7
Centre for social paediatrics	139.39	55.76	278.79	60.33	22.62	113.11	9.44	0.00	28.45	48.04	26.42	79.06	1.5
Psychiatric outpatient clinic	12.17	0.00	61.78	26.14	6.48	79.08	26.91	8.93	60.43	24.15	11.83	45.24	0.7
ASD outpatient clinic	425.83	292.00	580.76	355.87	254.89	473.20	225.43	168.32	293.06	301.10	249.71	360.19	9.2
Dentist/Orthodontist	131.06	100.98	168.26	190.62	156.08	233.11	102.64	85.53	123.06	135.48	120.25	154.37	4.1
Ophthalmologist/Optician	27.40	17.43	38.60	36.79	27.20	49.65	23.24	16.52	32.21	28.25	23.06	34.42	0.9
ENT specialist/hearing aid audiologist	14.90	6.91	33.83	8.01	3.30	19.62	13.44	8.72	19.58	11.96	8.63	17.00	0.4
Non-physician practitioner services													
Speech therapy	970.65	724.70	1250.92	252.69	146.70	412.92	94.76	41.85	188.97	290.78	220.67	371.89	8.8
Occupational therapy	1086.24	849.78	1321.17	294.61	188.01	430.93	170.17	104.56	266.02	363.90	292.94	448.30	11.1
Physiotherapist	170.81	94.27	285.03	151.65	98.54	222.17	129.92	85.31	198.72	143.83	111.40	186.83	4.4
Medication													
Prescription medication	114.10	51.84	243.30	299.94	206.34	444.07	492.98	352.32	939.23	365.87	288.58	588.51	11.1
Total	**4177.01**	3497.54	5000.22	**2509.88**	2065.93	3287.96	**3466.21**	2618.03	5071.10	**3286.86**	2811.50	4140.95	

In the regression model, female sex (Cost Ratio (CR): 1.64), lower IQ (CR: 1.90), and Asperger syndrome (CR: 1.54) were significantly associated with higher costs (Table 5).

**Table 5:** Cost ratios (with 95% CIs) using multivariate generalised linear regression

Predictor	Cost ratios	95% CI	
Sex			
Male	Reference		
Female	**1.65**	**1.13**	**2.37**
Age groups, in years			
0–11	Reference		
12–17	0.66	0.42	1.04
≥ 18	0.71	0.46	1.11
Diagnoses			
Childhood autism (F84.0)	Reference		
Atypical autism (F84.1)	1.37	0.90	2.09
Asperger syndrome (F84.5)	**1.54**	**1.03**	**2.32**
Other (F84.8/.9)	1.05	0.25	4.35
Intellectual functioning			
No learning disability/intellectual disability	Reference		
Learning disability/intellectual disability	**1.90**	**1.38**	**2.61**
ADOS comparison score			
Minimal to low (1–4)	Reference		
Moderate (5–7)	0.84	0.57	1.24
High (8–10)	0.78	0.52	1.19

## Discussion

The main results of this study are as follows:

1. When roughly comparing our findings to the few existing studies from Europe (Barrett et al., 2012, 2015; Roddy & O'Neill, 2019), and from the US (Lavelle et al., 2014), disregarding differences in study design, the ASD-related health costs found in this study are approximately in the medium range.
2. Regarding cost components, inpatient treatment had the largest share. Surprisingly, occupational therapy was a significant cost driver in children.
3. In terms of cost predictors, female sex was significantly associated with higher health costs, while ASD symptom severity was not.

### Health Costs

The number of existing questionnaire-based studies on health costs in individuals with ASD is very scarce, Table 6 provides an overview of the four relevant studies. As comparability with older studies may be hampered by differing diagnostic criteria (e.g. ICD-9 vs. ICD-10), divergent therapeutic guidelines, different reimbursement policies, or limited availability of autism-specific health services, the table contains only studies from the last ten years. The study of Xiong et al. (2011), while matching the general comparability criteria, was excluded because not enough details for reasonable comparison were reported.

**Table 6:** Overview of recent survey-based ASD health cost studies

Author	Country	Sample size	Age in years (mean, range)	Diagnoses	Health service costs	Influencing factors
This study	Germany	385	22.0 (4–67)	Clinically-diagnosed ASD (ICD-10: F84.0/.1/.5/.8/.9)	3287 EUR/12 months	Higher costs: Female sex, intellectual disability, Asperger syndrome No association: ASD severity, age
Roddy 2019 (Roddy & O'Neill, 2019)	Ireland	222	9.1 (2–18)	Parent-reported clinically-diagnosed ASD (DSM-5 criteria)	1355 EUR/12 months^a^	Higher costs: Comorbidity count, living in rural area No association: ASD severity, intellectual disability, sex, SES, maternal education
Barrett 2015 (Barrett et al., 2015)	UK	96	15.7	Clinically-diagnosed ASD (ICD-10: F84.0/1./.5/.8)	£500/6 months^b^	Higher costs: Lower age, lower adaptive functioning No association: Sex, ethnicity, ASD symptom scores, level of mental health difficulties
Lavelle 2014 (Lavelle et al., 2014)	US	109	3–17	Parent-reported ASD	3020 US$/12 months^c^	Not reported
Barrett 2012 (Barrett et al., 2012)	UK	152	2–5	Clinically-diagnosed ASD	£1383/6 months^d^	Higher (total) costs: Higher age, more ADI-R domains above cut-off No association: Sex, ethnicity, parental education, number of months since diagnosis, ADOS score

^a^State expenditure

^b^Includes hospital services, medication, and community health and social services

^c^The amount reported is the additional cost in comparison to patients without ASD

^d^This amount also includes community social and voluntary service

When comparing the cost figures from Table 6, it has to be kept in mind that there are considerable differences between the health systems of the respective countries. For example, while in Germany psychotherapy both for children and adults is broadly available and reimbursed by statutory health insurance funds, in the UK psychological therapies are less accessible (NHS England, 2018), with patients often paying out-of-pocket for private therapists. Also, the mental health system in the UK is designed with a much stronger focus on outpatient and/ or day-care treatment; in contrast to Germany, which is top of the table in Europe in terms of inpatient beds per 100,000 inhabitants (Signorini et al., 2017).

Moreover, study designs differ in terms of sample size, age range, diagnostic quality, included diagnostic categories, and cost composition.

In the study of Barrett et al. (2012), costs for healthcare, medication, and community social and voluntary services in UK toddlers with ASD were £1383/6 months, which is very close to our findings. As their sample only included 2- to 5-year old children, who usually do not use health services extensively, the similarity in cost sizes is probably due to substantial costs for nursery placement for most children in the Barrett et al. study.

In contrast, in the study of Barrett et al. (2015), costs (£500/6 months) were much lower than in our current study. This is somewhat surprising, as participants were on average 15.7 years old—an age, in which patients with ASD usually show a number of co-occurring conditions which need therapy. While costs for hospital services are roughly comparable to our findings, costs for outpatient (“community”) services are much lower than in our study, which may indicate a lower availability of these services in the UK health system.

In relation to the results of Roddy and O’Neill (2019) from Ireland, our cost figures are more than 2.4-fold higher, which may be due to the stronger focus on cheaper outpatient services within the Irish health system, and the restricted access to medical resources in this country (Thomas et al., 2014).

Finally, the “true” costs for children with ASD in the study by Lavelle et al. (2014) are most likely higher than our data, as their study only reported the *additional* costs for children with ASD in the US, i.e. the difference to health costs for children without an ASD diagnosis. In their sample, patients with an ASD diagnosis had significantly more outpatient visits, home healthcare visits, and prescription medication.

Remarkably, the average individual costs found in our questionnaire-based study come very close to the data from a cost-of-illness model for individuals from Germany with ASD and an IQ > 70 (0–18 years: 2473–3252 EUR, > 18 years: 3664 EUR) (Bachmann et al., 2013).

### Cost Components

In our study, costs for inpatient services were the largest cost component (over all age groups: 19.8% of total cost), accounting for 36.0% of total costs in adults with ASD. At least for psychiatric healthcare services in children with ASD, the preponderance of inpatient service cost has also been shown by other researchers (Croteau et al., 2019). The afore-mentioned cost structure is similar to the health cost distribution in schizophrenia, where inpatient costs also are the greatest component of health service costs in most countries (Kovács et al., 2020), including Germany (Konnopka et al., 2009). Yet, unlike in schizophrenia, inpatient costs in ASD are potentially mainly driven by the treatment of co-occurring psychiatric conditions. The rather comparable dimension of inpatient treatment costs in ASD and schizophrenia stands in contrast to the differences of these conditions in terms of stigma (much lower in ASD) and quality of life (much higher in ASD) levels (Bachmann et al., 2019; Kamp-Becker et al., 2010). In opposition to this, the composition of health costs for individuals with ADHD shows a higher share of outpatient costs compared to inpatient costs (Daley et al., 2020).

The fact that the overall cost shares of pharmacotherapy (11.1%) and occupational therapy (11.1%) are similar is somewhat surprising, given the shaky evidence for occupational therapy for ASD-related symptoms (usually sensory features) (Watling & Hauer, 2015). Even more surprising is the proportion of occupational therapy in children, namely 26.0%, despite this treatment modality not being recommended in this age group for the treatment of ASD core symptoms (Freitag et al., 2020; National Institute for Health & Care Excellence, 2013). Given the high popularity of occupational therapy in Germany also for other neurodevelopmental disorders in children and adolescents, e.g. ADHD (Braun et al., 2013), our findings seem to be a German peculiarity, which probably reflects the good availability of these services (in contrast to e.g. early intensive behavioural interventions) and prescribers’ lacking knowledge of evidence-based interventions for ASD. Nevertheless, in our opinion, occupational therapy constitutes a rather unfounded cost driver (Haverkamp et al., 2017).

### Cost Predictors

In this study, lower IQ, female sex, and Asperger syndrome were associated with higher health costs in individuals with ASD, while age or symptom severity (as indicated by the ADOS comparison score) were not. Regarding the association of lower IQ with higher costs, this result is in line with previous studies. The study of Buescher et al. (2014), for example, reported 1.6- to 1.7-fold greater lifetime costs for individuals with an ASD and intellectual disability (ID) vs. individuals with an ASD and no ID, which fits well with the cost ratio of 1.9 from our study.

The association of female sex with higher ASD health costs is a novel finding. One possible explanation are statistical effects—often, ASD studies suffer from low numbers of females (Reinders et al., 2019), therefore some gender-related effects may be difficult to detect. Another potential cause is higher psychiatric comorbidity in females with ASD, which may lead to higher healthcare costs through higher hospitalisation rates: In a German sample of adults with ASD, females had higher comorbidity rates (42%; vs. 30% in males) (Strunz et al., 2014). For children and adolescents, a Swedish study yielded similar results regarding symptom severity for comorbid ADHD, learning disabilities, and oppositional defiant disorder (Lundström et al., 2019). Yet, the existing literature regarding psychiatric comorbidity rates is not unequivocal: In a Swedish study of adults with ASD and normal intelligence (including a high portion of pervasive developmental disorder not otherwise specified), no difference between men and women in terms of lifetime psychiatric axis I diagnoses were found (Hofvander et al., 2009). Finally, the higher costs for females with ASD may be partly due to lengthy and cost-intensive diagnostic procedures, because making a correct diagnosis of ASD in females can be more complex and challenging than in males (Lundström et al., 2019; Ratto et al., 2018; Young et al., 2018). Of course, our finding needs to be replicated in a well-composed and reasonably sized sample of individuals with ASD.

The association of Asperger syndrome with higher costs is novel, too. A possible explanation for this finding is that mental health needs of other individuals with ASD, e.g. those with childhood autism and intellectual disability may be overlooked or misdiagnosed (example: signs of depression being interpreted by carers as ‘difficult behaviour’), thus leading to lower service use.

Interestingly, in our study age was not associated with costs. This stands in clear contrast to other studies which reported higher costs in older individuals (Barrett et al., 2015; Rogge & Janssen, 2019). A possible explanation are the high costs for occupational therapy and speech therapy for children in our sample. Finally, the lacking influence of ASD symptom severity (as reflected by the ADOS comparison score) is fully in line with the available literature so far (Barrett et al., 2012, 2015; Roddy & O'Neill, 2019).

### Strengths and Limitations

This study constitutes the first survey-based health cost study in adults with ASD, and is one of the few international studies that are based on real-world service use data. Moreover, the sample included a broad age range (spanning 4–67 years), and a significant proportion of females. To ensure high quality diagnoses, the diagnostic assessment of participants employed the current gold standard; which is key given the significant rates of over- and misdiagnoses in ASD (Bachmann et al., 2018).

A clear limitation of this study is the sample composition: This study is not a population-based study, but a survey of patients of four large German ASD outpatient clinics. While the patient population of these clinics is representative for a typical clinical population of individuals with ASD in Germany in terms of geography (the patients seen in the four study centres stem from all German federal states, with the exception of Mecklenburg-Vorpommern), age, male/female ratio, IQ, and level of ASD symptom severity), it only contains those service users with a formal ASD diagnosis.

Moreover, the above-average portion of individuals with Asperger syndrome in our sample may have led to a bias in health costs distribution.

Another limitation is the lack of information on co-occurring health conditions, on adaptive functioning (Szatmari et al., 2015), and on participants’ socio-economic status, which probably are important service use (Drahota et al., 2020) and cost predictors in addition to the predictors studied in this work. Also, as the CSRI time frame included service use in the previous 12 months, a recall bias is possible.

While health costs are an important cost component in ASD-related (especially regarding the potentially higher morbidity and mortality), other cost types, e.g. educational costs, can be more important cost drivers in ASD-related societal costs (Bachmann et al., 2013; Rogge & Janssen, 2019). Finally, due to differences in health systems between countries, generalisability of our results is limited.

## Conclusion

Compared to the few existing studies, the ASD-related health costs found in this study are in the medium range. The level of ASD-related health costs was comparable to those of individuals with schizophrenia, thus underlining the public health relevance of ASD. The considerable share of occupational therapy costs is surprising, given the lacking evidence for the effectiveness of this method in the treatment of ASD-related symptoms. The findings regarding higher ASD health costs in females are novel and require replication.

## Supplementary Information

Below is the link to the electronic supplementary material.

Supplementary Information 1 (DOCX 30 KB)

## Data Availability

All data generated or analysed during this study are included in this published article.
