# Supplementary material for: Health Services Use and Costs in Individuals with Autism Spectrum Disorder in Germany: Results from a Survey in ASD Outpatient Clinics
Source: J Autism Dev Disord. 2021 Mar 17;52(2):540–52. doi: 10.1007/s10803-021-04955-4 (PMC8813793; doi:10.1007/s10803-021-04955-4)
Supplement: Supplementary file 1 — Supplementary Information 1 (DOCX 30 KB) [file 10803_2021_4955_MOESM1_ESM.docx]

***Supplement S1. Detailed data sources.***

| **Type of service** | **Unit** | **Unit costs (source for monetary valuation)** | | | **Price per unit (2016)** | | | |  |  |
| --- | --- | --- | --- | --- | --- | --- | --- | --- | --- | --- |
| **Inpatient services** |  |  | | |  | |  | |  |  |
| Psychiatric or psychosomatic inpatient care | Days | Mean costs of care per day (1–5) | | | | 524.32 | EUR | |  |  |
| Non-psychiatric inpatient care | Days | Mean costs of care per day (1–5) | | | | 227.94 | EUR | |  |  |
| Inpatient rehabilitation centre | Days | Mean costs of care per day (1,2,6) | | | | 154.74 | EUR | |  |  |
| **Other hospital-based services** |  |  | | | |  |  | |  |  |
| Emergency department | Days | Median revenue per KV^1^ emergency (7) | | | | 34.60 | EUR | |  |  |
| Outpatient clinic (for adults) | Days | Mean of different EBM-Codes^2^ for *Ermächtigungsambulanzen*^3^ for adults (8^a^) | | | | 115.83 | EUR | |  |  |
| Outpatient clinic (for children) | Days | Mean of different EBM-Codes^2^ for *Ermächtigungsambulanzen*^3^ for children (8^a^) | | | | 50.30 | EUR | |  |  |
| Day care unit | Days | Mean cost of care per day (1–5) | | | | 148.16 | EUR | |  |  |
| **Outpatient services** |  |  | | | |  |  | |  |  |
| General practitioner | Contacts | Mean costs per contact (1,9–12) | | | | 21.17 | EUR | |  |  |
| Paediatrician | Contacts | Mean costs per contact (1,9–12) | | | | 36.17 | EUR | |  |  |
| Adult psychiatrist | Contacts | Mean costs per contact (1,9–12) | | | | 45.46 | EUR | |  |  |
| Child and adolescent psychiatrist | Contacts (max. 4) | Fixed amount for social psychiatry and mean of different EBM-Codes^2^ (8^a^) | | | | 215.80 | EUR | |  |  |
| Psychotherapist (for adults) | Contacts | Mean costs per contact (1,9–12) | | | | 81.61 | EUR | |  |  |
| Psychotherapist (for children) | Contacts | Mean of different EBM-Codes^2^ for psychotherapy for children (8^a^) | | | | 87.25 | EUR | |  |  |
| Centre for social paediatrics | Quarter (max. 4) | Quarterly fixed amount for a center for social paediatrics (SPZ^4^ Oldenburg) | | | | 460.00 | EUR | |  |  |
| Psychiatric outpatient clinic (for adults) | Quarter (max. 4) | Mean quarterly fixed amount for psychiatric outpatient clinics for adults (PIA^5^) (13^b^) | | | | 291.56 | EUR | |  |  |
| Psychiatric outpatient clinic (for children) | Quarter (max. 4) | Mean quarterly fixed amount for psychiatric outpatient clinics for children (KJPIA^6^) (13^b^) | | | | 395.41 | EUR | |  |  |
| ASD outpatient clinic (for adults) | Quarter (max. 4) | Mean quarterly fixed amount for psychiatric outpatient clinics for adults (PIA^5^) (13^b^) | | | | 291.56 | EUR | |  |  |
| ASD outpatient clinic (for children) | Quarter (max. 4) | Mean quarterly fixed amount for psychiatric outpatient clinics for children (KJPIA^6^) (13^b^) | | | | 395.41 | EUR | |  |  |
| Dentist/Orthodontist | Contacts | Mean costs per contact (1,14,15) | | | | 51.32 | EUR | |  |  |
| Ophthalmologist/Optician | Contacts | Mean costs per contact (1,9–12) | | | | 40.47 | EUR | |  |  |
| ENT specialist/Hearing aid audiologist | Contacts | Mean costs per contact (1,9–12) | | | | 28.49 | EUR | |  |  |
| **Non-physician practitioner services** |  |  | | | |  |  | |  |  |
| Speech therapy | Contacts | Mean costs per contact (1,16) | | | | 41.43 | EUR | |  |  |
| Occupational therapy | Contacts | Mean costs per contact (1,16) | | | | 39.41 | EUR | |  |  |
| Physiotherapist | Contacts | Mean costs per contact (1,16) | | | | 17.85 | EUR | |  |  |
| **Medication** |  |  |  | | | | | |  |  |
| Prescription medication | DDD^7^ | Mean costs of defined daily doses (17) | | Costs vary by specific medicine and participant’s age | | | | |  |  |
|  |  |  | |  | | | | |  |  |
|  |  |  | |  | | | | |  |  |
| ***Annotations:*** | | | | | | | |  | |  |
| *^1^ KV (Kassenärztliche Vereinigung, Association of Statutory Health Insurance Physicians)*  *^2^ EBM-Codes (Einheitlicher Bewertungsmaßstab, Doctors’ Fee Scale within the Statutory Health Insurance Scheme)* | | | | | | | |  | |  |
| *^3^ Ermächtigungsambulanzen (Specially-entitled outpatient departments)* | | | | | | | |  | |  |
| *^4^ SPZ (Sozialpädiatrisches Zentrum, Centre for Social Paediatrics)*  *^5^ PIA (Psychiatrische Institutsambulanz, Hospital-associated psychiatric outpatient clinic)*  *^6^ KJPIA (Psychiatrische Institutsambulanz für Kinder und Jugendliche, Hospital-associated psychiatric outpatient clinic for children and adolescents)*  *^7^ DDD (defined daily doses)*  *^a^ Selection of EBM-Codes was made by specialists in the respective field.*  *^b^ In addition to the fixed amounts for PIAs from Lower Saxony, fixed amounts from PIAs in Berlin and Hassia were included in cost modeling.* | | | | | | | |  | |  |

***References:***

*1. Bock JO, Brettschneider C, König HH, Seidl H, Holle R, Bowles D, et al. Calculation of standardised unit costs from a societal perspective for health economic evaluation. Gesundheitswesen. 2015;*

*2. Bundesministerium für Gesundheit. Gesetzliche Krankenversicherung. Endgültige Rechnungsergebnisse 2016. 2017.*

*3. Bundesministerium für Gesundheit. Gesetzliche Krankenversicherung - Kennzahlen und Faustformeln. 2019.*

*4. Statistisches Bundesamt. Gesundheit. Kostennachweis der Krankenhäuser 2016. Fachserie 12 Reihe 6.3. 2017.*

*5. Statistisches Bundesamt. Gesundheit. Grunddaten der Krankenhäuser 2016. Fachserie 12 Reihe 6.1.1. 2017.*

*6. Bundesministerium für Gesundheit. Ergebnisse der Statistik KG 5, Vorsorge- und Rehabilitationsmaßnahmen 2016 der Gesetzlichen Krankenversicherungen. 2017.*

*7. Blum K, Löffert S, Offermanns M, Steffens P. Krankenhaus Barometer. Umfrage 2017. Deutsches Krankenhausinsitut; 2017.*

*8. Kassenärztliche Bundesvereinigung. Einheitlicher Bewertungsmaßstab (EBM) Stand: 1. Quartal 2016. 2016.*

*9. Kassenärztliche Bundesvereinigung. Grunddaten zur vertragsärztlichen Versorgung in Deutschland 2008.*

*10. Bundesministerium für Gesundheit. KM6-Statistik (gesetzliche Krankenversicherung: Versicherte) zum 01. Juli 2007.*

*11. Riens B, Erhart M, Mangiapane S. Arztkontakte im Jahr 2007. Zentralinstitut für die kassenärztliche Versorgung in Deutschland. Versorgungsatlas-Bericht Nr 12/02. 2012;*

*12. Kassenärztliche Bundesvereinigung. Kennzahlen der Abrechnungsgruppen. Honorarbericht 1. Quartal 2009 - 4. Quartal2016. 2018.*

*13. Niedersächsische Krankenhausgesellschaft. Psychiatrische Institutsambulanz (PIA). Vergütung in Niedersachsen [Internet]. 2019. Available from: https://www.nkgev.info/PIA.html*

*14. Rädel M, Bohm S, H-W P, Walter M. BARMER Zahnreport 2018. Schriftenreihe zur Gesundheitsanalyse. 2018.*

*15. Kurt P. Krankheitskostenanalyse der frühen juvenilen idiopathischen Arthritis - nach aktuellem Versorgungsstandard. Dissertation. 2018.*

*16. GKV-Spitzenverband. Heilmittel-Schnellinformation. Bundesbericht Januar bis März 2016. 2016.*

*17. Schwabe U, Paffrat D, Ludwig W-D, Klauber J. Arzneiverordnungs-Report 2017. Springer Berlin Heidelberg; 2017.*

**Corresponding author:**

Christian J. Bachmann, MD, PhD

Department of Child and Adolescent Psychiatry

Universitätsklinikum Ulm

Steinhövelstr. 5, 89075 Ulm, Germany

Phone: +49 731 500-61603

Fax: +49 731 500-61602

E-mail: christian.bachmann@uniklinik-ulm.de
